# Emerging small-molecule antiviral agents in long COVID prevention

**DOI:** 10.3389/fphar.2024.1457672

**Published:** 2024-10-09

**Authors:** Xiaomeng He, Xiang Zhang, Wu Zhong

**Affiliations:** ^1^ National Engineering Research Center for the Emergency Drug, Beijing Institute of Pharmacology and Toxicology, Beijing, China; ^2^ Department of Blood Transfusion Medicine, The 940th Hospital of the Joint Logistics Support Force of the Chinese People’s Liberation Army, Lanzhou, China

**Keywords:** long COVID, metformin, ensitrelvir, molnupiravir, nirmatrelvir

## Abstract

Long COVID, or Post-Acute Sequelae of COVID-19 (PASC), was characterized by persistent symptoms such as fatigue, shortness of breath, and cognitive impairments. These symptoms, emerging one to 2 months post-infection and persisting for several months, cannot be attributed to other diagnoses. The pathophysiology of long COVID remained elusive; however, emerging studies suggested multiple potential mechanisms, including the reactivation of Epstein-Barr virus, persistent SARS-CoV-2 reservoirs, neuroinflammation, and vascular damage, which may contribute to its development. Long COVID affected multiple organ systems, including respiratory, circulatory, and nervous systems, leading to a range of functional impairments. Additionally, it showed a profound impact on mental health, manifesting as anxiety and depression, which significantly degraded the quality of life. The absence of definitive treatments underscored the importance of prevention. Recent evidence indicated that early antiviral intervention—particularly with small-molecule drugs such as Metformin, Ensitrelvir, Molnupiravir, and Nirmatrelvir—may effectively reduce the incidence of long COVID. This underscored the promising role of small-molecule compounds in mitigating long-term COVID-19 consequences, offering a novel preventive strategy against long COVID and its extensive impacts on patients.

## 1 Introduction

Long COVID, a chronic medical condition recognized under various synonyms including Post-Acute Sequelae of COVID-19 (PASC) and Post-COVID-19 condition, was officially classified in the International Classification of Diseases (ICD-10) on 1 October 2021, with current diagnosis code RA02 (McGrath et al., 2022; World Health Organisation, 2024). This condition, which recently gained recognition, was subject to ongoing definitional debates. The World Health Organization (WHO) described long COVID as manifesting approximately 3 months from the onset of COVID-19 with symptoms persisting for at least 2 months, without explanation by alternative diagnoses. Symptoms commonly included fatigue, cognitive dysfunction, and others, which may arise anew following initial recovery or persist from the initial infection (World Health Organisation, 2024). The National Institutes of Health (NIH) in the United States categorized long COVID as symptoms that were persistent, new, or recurring more than 4 weeks after initial SARS-CoV-2 infection (National Institutes of Health, 2024). Similarly, the United Kingdom National Institute for Health and Care Excellence (NICE) defined it as a multisystem condition with a variety of debilitating symptoms that continued or developed after acute COVID-19, persisted for more than 4 weeks, and cannot be attributed to another diagnosis (Altmann et al., 2023). There were many differences in the definition of these organizations, such as time of onset, persisting duration and symptoms, etc. These variations in definition reflected the heterogeneity and complex etiology of long COVID, leading to concerns about the specificity and sensitivity of its clinical characterization (Merad et al., 2022; Davis et al., 2023). A more precise definition awaited further clinical evidence and detailed investigation.

## 2 Increasing symptoms and potential mechanisms of long COVID

A prospective study named RECOVER, as outlined in Table 1, was initiated to define PASC more precisely using an array of symptoms including post-exertional malaise (PEM), fatigue, cognitive impairments (often referred to as “brain fog”) (Becker et al., 2021), dizziness, gastrointestinal reactions, palpitations, changes in sexual desire or capacity, alterations in smell or taste, increased thirst, chronic cough, chest pain, and abnormal movements (Thaweethai et al., 2023). These 12 symptoms of PASC were ranked by their frequency of occurrence from highest to lowest. The RECOVER study was notable as a convincing prospective cohort study that included an uninfected comparison group, a feature lacking in most retrospective clinical cohort studies. Crucially, this research systematized the definition of long COVID around these 12 symptoms, establishing a foundation for more specific and actionable clinical definitions.

**TABLE 1:** Participant selection for RECOVER and main outcomes.

Participants: 9,764 adults (71% female; 16% hispanic/Latino; 15% non-hispanic black; 58% fully vaccinated at the index date; median age 47 years) i. Enrolled in the RECOVER adult cohort before 10 April 2023 ii. Completed a symptom survey 6 months or more after acute symptom onset or test date							
	Frequency						
	PASC (%)	Post-exertional malaise (%)		Fatigue (%)		Brain fog	
Infected (8,646)	23	28	21% (absolute difference in frequencies)	38	21% (absolute difference in frequencies)	20%	16% (absolute difference in frequencies)
Uninfected (1,118)	3.7	7		17		4%	

The etiology of long COVID was complex due to its broad spectrum of symptoms, suggesting multiple overlapping causes. Recent studies revealed the pathophysiology of PEM. The skeletal muscle structure of patients with long COVID would change, including metabolic disorders in skeletal muscle, severe exercise induced myopathy and tissue infiltration containing amyloid deposits, thus inducing the deterioration of PEM (Appelman et al., 2024). American researchers proposed a potential shared mechanism between PASC symptoms—such as fatigue, post-exertional malaise, and cognitive dysfunction (commonly termed as ‘brain fog’)—and myalgic encephalomyelitis/chronic fatigue syndrome (ME/CFS) (Davis et al., 2023). ME/CFS, a multifaceted disorder characterized by severe, persistent fatigue lasting at least 6 months, was acknowledged as a multisystem neuroimmune condition often triggered by viral infections (Rasa et al., 2018). Viruses commonly associated with ME/CFS included Epstein–Barr virus (EBV), *Coxiella* burnetii (the causative agent of Q fever), Ross River virus, and West Nile virus (Choutka et al., 2022). Furthermore, emerging clinical studies linked EBV reactivation with the fatigue observed in long COVID, evidenced by an increase in EBV antibodies in patients, suggesting that this reactivation might play a role in long COVID pathogenesis (Klein et al., 2023; Peluso et al., 2023).

Studies focusing on the neuropathological mechanisms of cognitive impairment in long COVID identified potential causes such as neuroinflammation, vascular damage, and neuronal injury (Spudich and Nath, 2022). Autopsies of patients who succumbed to COVID-19 revealed widespread microthrombi—both obstructive and non-obstructive—in brain blood vessels, accompanied by neuronal loss and microglial phagocytosis (Lee et al., 2021; Lee et al., 2022). Recent study provided new evidence that the blood-brain barrier of patients with long COVID and brain fog was more permeable than that of uninfected people. Therefore, in addition to inflammatory factors, the leakage of blood-brain barrier led to some cognitive symptoms of long COVID (Greene et al., 2024).

## 3 Small-molecule compounds for long COVID prevention

**FIGURE 1:**
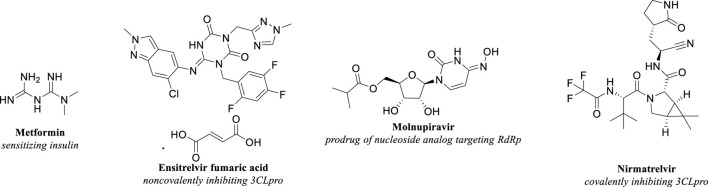
The molecular formulas of Metformin, Ensitrelvir, Molnupiravir, and Nirmatrelvir.

While the precise definition and underlying mechanisms of long COVID continued to be subjects of extensive research, identifying effective strategies to mitigate this condition remained critically important. In addition to preventive measures such as avoiding infection and vaccination, evidence-based approaches emphasized the significance of early antiviral interventions, including Metformin, Ensitrelvir, Molnupiravir, and Nirmatrelvir. Administering antiviral medications at the onset of COVID-19 was suggested to help prevent the development of long COVID. The molecular structures of these compounds were depicted in Figure 1.

### 3.1 Metformin

Metformin, a small-molecule drug renowned for its insulin-sensitizing properties, was typically the first-line treatment for type 2 diabetes. It operated by phosphorylating and activating AMP-activated protein kinase (AMPK) in hepatocytes, improving glucose and lipid metabolism (Sharma et al., 2020). Additionally, Metformin influenced the gut microbiome and enhanced the excretion of glucose into feces (Wu et al., 2017; Morita et al., 2020). Beyond these effects, Metformin was associated with weight reduction through increased levels of the peptide hormone growth/differentiation factor 15 (GDF15), which decreased food intake (Gerstein et al., 2017; Coll et al., 2020). It also was found to reduce the frequency of various cancers by inhibiting the mechanistic target of rapamycin (mTOR) via AMPK activation in pre-neoplastic cells, thereby hindering cell proliferation and promoting apoptosis (Chan, 2016; Demb et al., 2019; Kim et al., 2020). As a conclusion, in addition to the classic antihyperglycemic properties, Metformin improved gut microbiome, reduced food intake, and showed anti-tumor effects. In recent years, some clinical data showed that it probably had antiviral effects.

During the COVID-19 pandemic, Metformin had exhibited promising effects for patients with diabetes (Penlioglou et al., 2020; Zhu et al., 2020). Early observations in 2020 from a study involving 309 patients with both type 2 diabetes and COVID-19 revealed that those treated with Metformin had a mortality rate of 8.7%, notably lower than the prevailing COVID-19 mortality rates at the time (Shestakova et al., 2020). We all know that Metformin reduced mortality related to diabetes, and this Russian team was the first reporting trial in patients with both diabetes and COVID-19; The Metformin group had a mortality rate of only 8.9%, indicating that it also reduced COVID-related mortality then. Further evidence from a large-scale clinical trial indicated that regular Metformin treatment correlated with a better prognosis in COVID-19 patients, demonstrating decreased mortality risk and increased likelihood of discharge within 28 days (Wargny et al., 2021). These findings suggested that Metformin may act as an antiviral agent against SARS-CoV-2, although the precise mechanisms warranted further experimental exploration (Chen et al., 2023).

**FIGURE 2:**
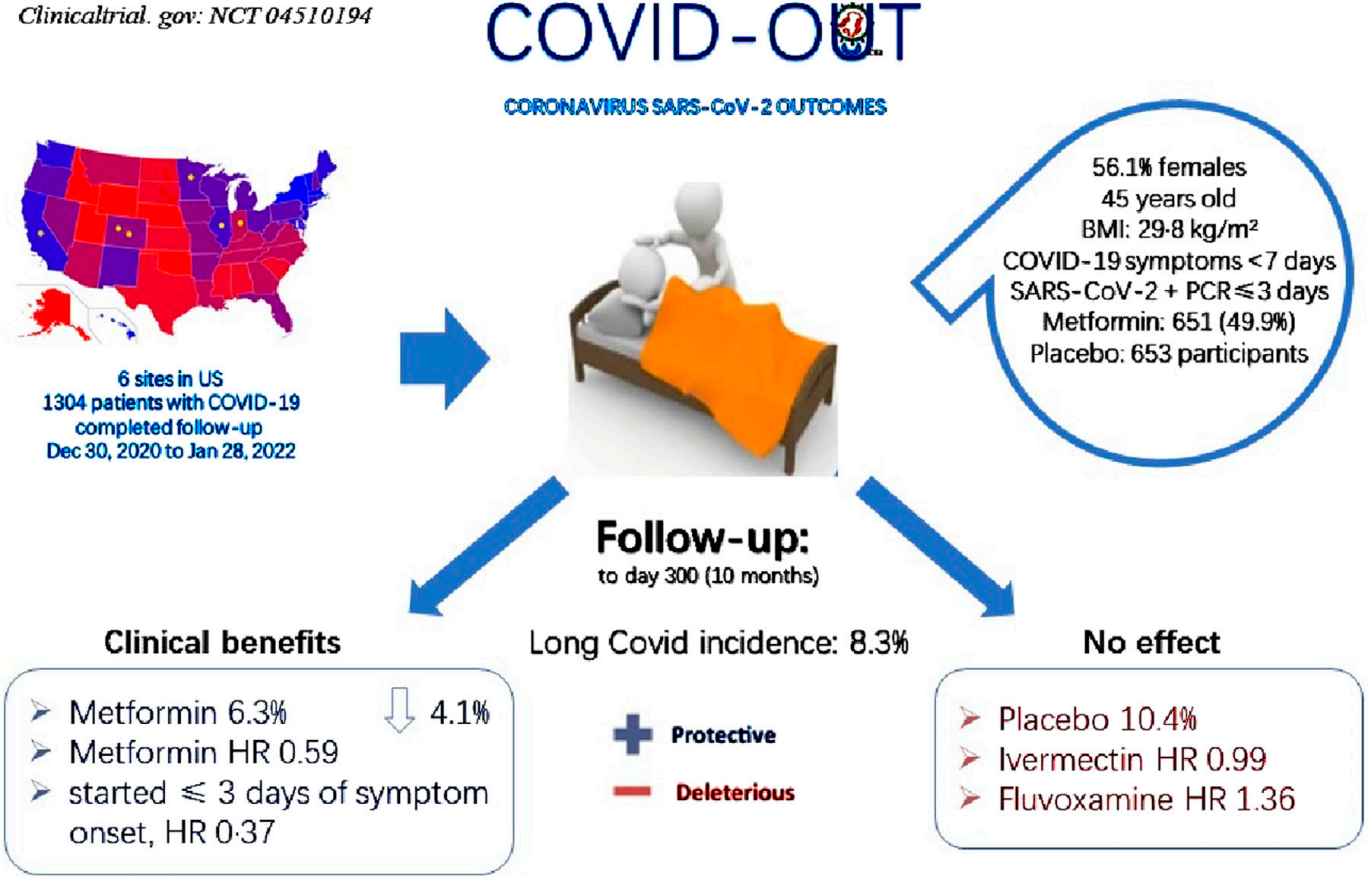
Participant selection for COVID-OUT and main outcomes. Compared with placebo group 10.4%, Metformin group showed 6.3% long COVID incidence, decreasing 4.1% with protective effect from long COVID; Divided the overall long COVID incidence 8.3%, HR 0.59 was calculated; Compared with Ivermectin HR 0.99 as well as Fluvoxamine HR 1.36, Metformin showed obviously protective effect while Ivermectin and Fluvoxamine showed deleterious effect.

A Phase 3 clinical trial, COVID-OUT, testing Metformin among other drugs for preventing long COVID, reported breakthrough results (Figure 2) (Bramante et al., 2023). Enrolling 1,304 participants, all COVID-19 patients, the trial found that Metformin usage yielded a hazard ratio (HR) of 0.59 for preventing long COVID compared to placebo (95% CI 0.39–0.89; *p* = 0.012), indicating a 41% relative reduction in long COVID incidence. When initiated within 3 days of COVID-19 onset, the hazard ratio was further reduced to 0.37, underscoring the effectiveness of early Metformin intervention. These outcomes not only highlighted the potential of early Metformin administration in preventing long COVID but also affirmed its substantial efficacy, supporting its use in clinical practice for managing long COVID. Additionally, reports suggested that Metformin may alleviate long COVID-associated tinnitus, further endorsing its role in reducing the occurrence of long COVID symptoms (Kelleni, 2024).

### 3.2 Ensitrelvir

Ensitrelvir was an oral small-molecule anti-COVID-19 drug approved by the Ministry of Health, Labour, and Welfare in Japan on 5 March 2024 (Shionogi and Co, 2024a). It worked by noncovalently inhibiting the SARS-CoV-2 3CL Protease, a viral protease crucial for viral replication. This inhibition prevented the formation of essential enzymes for replication, such as RNA-dependent RNA polymerase (RdRp) (Unoh et al., 2022).

Recently, Shionogi company provided updates from a global phase 3 study of Ensitrelvir (SCORPIO-HR) for non-hospitalized participants with COVID-19, in which Ensitrelvir did not demonstrate a statistically significant reduction in the proportion of participants with long COVID at 3 months compared to placebo (Shionogi and Co, 2024b). However last year, in SCORPIO-SR, a pivotal phase III clinical trial, additional exploratory data on the risk reduction of long COVID in patients with mild to moderate COVID-19 recruited in Japan, South Korea, and Vietnam were released (Table 2). Ensitrelvir Fumaric Acid treatment was initiated within 5 days from COVID-19 onset. At 3 and/or 6 months, long COVID symptoms were assessed (Shionogi and Co, 2023). Among a subset of patients who reported high scores on 14 COVID-19 symptoms, the incidence of long COVID was 14.5% with 125 mg Ensitrelvir and 26.3% with placebo, representing a 45% relative risk reduction (RRR, *p* < 0.05); The incidence of the neurological symptoms of long COVID was 29.4% *versus* 44.0%, representing a relative risk reduction of 33% (*p* < 0.05), which illustrated that the use of Ensitrelvir at the early stage of COVID-19 onset effectively prevented long COVID in patients who reported high scores on 14 CVOID-19 symptoms. Based on the above two clinical trials, it was concluded that Ensitrelvir was effective at preventing long COVID in patients with more severe, compared to milder, COVID-19.

**TABLE 2:** Participant selection for SCORPIO-SR and main outcomes.

Patients: 1230 i. Mild/moderate COVID-19 ii. Predominantly (more than 90%) COVID-19 vaccinated iii. Reported high scores on 14 COVID-19 symptoms				
	Long COVID		PASC neurological symptoms	
	Incidence (%)	Relative risk reduction (%)	Incidence (%)	Relative risk reduction (%)
Ensitrelvir	14.5	45	29.4	33
Placebo	26.3		44.0	

### 3.3 Molnupiravir

Molnupiravir was the oral prodrug of *β-D*-N4-hydroxycytidine (NHC), which received Emergency Use Authorizations (EUA) from the FDA for the treatment of COVID-19. The uptake of NHC by viral RdRp led to viral mutations and lethal mutagenesis (Kabinger et al., 2021).

United States researchers examined Molnupiravir and the risk of PASC through a cohort study (Table 3). A total of 229,286 COVID-19 patients between 5 January 2022, and 15 January 2023, in the United States. Department of Veterans Affairs medical database, who had at least one risk factor for progression to severe COVID-19 and survived within the first 30 days after testing positive, were enrolled. 11,472 participants were prescribed Molnupiravir within 5 days of a positive result, while 217,814 participants received no antiviral or antibody treatment. Compared with the non-treatment control group, the use of Molnupiravir within 5 days of a positive SARS-CoV-2 test was associated with a reduced risk of developing long COVID. The relative risk (RR) was 0.86 (95% CI, 0.83–0.89), and the absolute risk reduction (ARR) was 2.97% (95% CI, 2.31%–3.60%) (Xie et al., 2023b).

**TABLE 3:** Patients selection and main findings.

Patients: 229,286 i. 2022.1.5–2023-1.15 in the United States. Department of veterans affairs medical database ii. At least 1 risk factor for progression to severe COVID-19 iii. Survived within the first 30 days after testing positive			
	Long COVID		
	Incidence (%)	Relative risk	Absolute risk reduction (ARR) (%)
Molnupiravir (11,472)	18.58	0.86	2.97
No treatment group (217,814)	21.55		

To conclude, in COVID patients with at least one risk factor for progression to severe COVID-19, the use of Molnupiravir within 5 days from SARS-CoV-2 infection may be a viable approach to reduce the risk of PASC, with relative risk reduction (RRR) 14% [(21.55–18.58)/21.55 = 0.1378].

### 3.4 Nirmatrelvir

Nirmatrelvir was an orally bioavailable protease inhibitor targeting M^PRO^, the same drug target as Ensitrelvir. The FDA had issued an Emergency Use Authorization (EUA) for Ritonavir-boosted Nirmatrelvir for the treatment of mild to moderate COVID-19.

Investigators as in the above Molnupiravir cohort study herein utilized the same United States. Department of Veterans Affairs medical database to identify individuals who tested positive for SARS-CoV-2 between 3 January 2022, and 31 December 2022 (Table 4). These individuals were not hospitalized on the day of their positive test, had at least one risk factor for developing severe COVID-19, and survived the first 30 days of SARS-CoV-2 infection (Xie et al., 2023b). They identified patients who received oral Nirmatrelvir within 5 days after positive test (n = 35,717) and those who did not receive COVID-19 antivirals or antibodies during the acute phase of SARS-CoV-2 infection (control group, n = 246,076). They calculated the effect of Nirmatrelvir (compared with the control group) on 13 long COVID symptoms at 180 days. The hazard ratio for Nirmatrelvir compared to the control group in preventing long COVID was 0.74 (95% CI, 0.72–0.77), with an absolute risk reduction (ARR) of 4.51% (95% CI, 4.01–4.99) as well as RRR 26% [(17.51–12.99)/17.51 = 0.2581]. This also led to a decreased risk of 10 long COVID symptoms, including dysrhythmia and ischemic heart disease in the cardiovascular system, deep vein thrombosis and pulmonary embolism in coagulation and hematologic disorders, fatigue and malaise, acute kidney disease, muscle pain, neurocognitive impairment, and shortness of breath. In particular, it was ultimately concluded that in SARS-CoV-2 infected patients with at least one risk factor for progression to severe disease, treatment with Nirmatrelvir within 5 days of a positive SARS-CoV-2 test was associated with a reduced risk of PASC, regardless of vaccination status and previous infection history (Xie et al., 2023a).

**TABLE 4:** Patients selection and main findings.

	Long COVID			Patients: 281, 793 i. Tested positive for SARS-CoV-2 between 2022.1.3–2022-12.10 in the United States. Department of veterans affairs medical database ii. Not hospitalized on the day of their positive test iii. At least 1 risk factor for progression to severe COVID-19 iv. Survived the first 30 days of sars-cov-2 infection		
	Event rate (%)	Hazard ratio	ARR			
Nirmatrelvir treatment (35,717)	12.99	0.74	4.51			
No treatment control group (246,076)	17.51					
				Hazard ratio	Event rate at 180 days after tested positive	
					Nirmatrelvir treatment	Control
Cardiovascular		Dysrhythmia		0.73	3.63	5.36
		Ischemic heart disease		0.71	1.58	2.65
Coagulation and hematologic		Deep vein thrombosis		0.72	0.3	0.52
		Pulmonary embolism		0.61	0.52	1.08
general symptoms		Fatigue and malaise		0.79	4.52	6.45
Gastrointestina		Liver disease		0.91	1.67	2.35
Kidney		Acute kidney injury		0.67	0.94	1.54
Musculoskeletal		Muscle pain		0.65	1.79	2.61
neurological symptoms		Neurocognitive impairment		0.74	1.61	2.51
		Dysautonomia		0.86	1.25	1.46
respiratory symptoms		Shortness of breath		0.89	5.35	6.54
		Cough		0.96	4.62	5.1
Metabolic		Diabetes		0.98	1.77	2.58

But there were also conflicting conclusions. In a simultaneously conducted observational cohort study within the Covid Citizen Science (CCS) study, a large online cohort study, researchers explored whether treatment with Nirmatrelvir during acute SARS-CoV-2 infection reduced the risk of long COVID. Vaccinated, non-hospitalized, and non-pregnant individuals who reported their first SARS-CoV-2 positive test result from March to August 2022 were selected. 353 treated and 1,258 untreated subjects responded to the long COVID survey (n = 1,611). At 5.4 ± 1.3 months post-infection, Nirmatrelvir treatment was not found to be associated with subsequent long COVID symptoms after adjustment for propensity (OR: 1.15; 95% CI: 0.80–1.64; *p* = 0.45) (Durstenfeld et al., 2024). This study found no association between Nirmatrelvir treatment and reduced risk of long COVID symptoms, contradicting other findings, which indicated that further in-depth research was needed to determine whether Nirmatrelvir treatment could reduce the risk of long COVID.

In another Molnupiravir cohort study conducted in Italy between April 2021 and March 2022 (Table 5), 649 COVID-19 patients were enrolled if they met the following criteria: (i) adults with positive SARS-CoV-2 nasopharyngeal swabs, (ii) reported mild/moderate COVID-19 symptoms onset within 5 days (oral antiviral drug) or 7 days (intravenous remdesivir or monoclonal antibody), and (iii) had at least one risk factor for COVID-19 progression (Bertuccio et al., 2023). Long COVID data at 3 months were available for 323 of these patients, with symptoms categorized as follows: general symptoms (tiredness or fatigue that interfered with daily life and fever); respiratory and heart symptoms (dyspnea, cough, pharyngodynia, chills, nasal congestion, chest pain, and heart palpitations); neurological symptoms (headache, lightheadedness, muscle pain, change in smell or taste); neurobehavioral symptoms (memory and concentration deficit, sleep problems, depression, or anxiety); gastrointestinal symptoms (vomiting, diarrhea). There were 77 patients in the small-molecule antiviral drug treatment group and 105 patients in the control group. In the small-molecule antiviral drug treatment group, 35 patients were treated with Molnupiravir, 32 patients were treated with Remdesivir, and 10 patients were treated with Ritonavir-Boosted Nirmatrelvir.

**TABLE 5:** Patients selection and main findings.

Patients: 649 i. Between April 2021 and March 2022 COVID-19 patients ii. Adults with positive SARS-CoV-2 nasopharyngeal swabs iii. Reported mild/moderate COVID-19 symptoms onset within 5 or 7 days iv. Presence of at least one risk factor for COVID-19 progression				
	Long COVID		PASC neuro-behavioural symptoms	
	Percentage	Odds ratio (OR)	Percentage	Odds ratio (OR)
Antiviral drugs (77)	20.8	0.43	18.2	0.51
Control (105)	34.3		25.7	
			Percentages of symptoms at 3 months after the onset of SARS-CoV-2 infection	
			Antivirals Treatment (N = 77)	Control (N = 105)
general symptoms	Fever		0.00	0.00
	Fatigue		14.30	21.90
respiratory and heart symptoms	Dyspnea		5.20	5.70
	Cough		0.00	2.90
	Pharyngodynia		0.00	0.00
neurological symptoms	Myalgia		1.30	3.80
	Ageusia/Dysgenusia		1.30	4.80
	Anosmia		0.00	1.90
	Headache		1.30	3.80
Neuro-behavioural symptoms (memory and concentration deficit, sleep problems and depression or anxiety)			7.80	8.60
Gastrointestinal symptoms (vomiting, diarrhea)			1.30	4.80

The survey found that the incidence of long COVID in the control group was higher than in the small-molecule antiviral drug treatment group. Compared with the control group, the early use of antiviral drugs showed an odds ratio (OR) of 0.43 (95% CI, 0.21–0.87).

## 4 Conclusion and outlook

By analyzing the mentioned findings, we discovered that among the four small molecule antiviral drugs, Metformin had the lowest hazard ratio (HR) value. Specifically, the hazard ratio (HR) for Metformin was 0.59 when taken within 7 days of infection and 0.37 when taken within 3 days of infection, while the values of the other three antiviral drugs were as follows: Ensitrelvir 0.55, Molnupiravir 0.86, and Nirmatrelvir 0.74. Therefore, taking Metformin during the infection period may have the best effect on preventing long COVID, and the earlier it was taken during the infection period, the better.

Secondly, in the aforementioned evidence, the studies on Metformin and Ensitrelvir were prospective placebo-controlled clinical Phase III trials, while the research on Molnupiravir and Nirmatrelvir only consisted of observational retrospective cohort studies; although both of which companies said they would continue to monitor clinical trial participants for 6 months after treatment (Ledford, 2022), as far no paper has published the clinical trial results.

Thirdly, the four drugs might have varying effects on different PASC symptoms. Observational retrospective cohort study of Nirmatrelvir had the most significant effect in preventing pulmonary embolism, with a hazard ratio (HR) value of 0.61 (Table 4), while others did not disclose effect on pulmonary embolism. In addition, this study listed in detail a good preventive effect on brain fog, while such effects of the other three antiviral drugs had not disclosed. It should be pointed out that due to lack of a universally accepted definition of long COVID, clinical trials and studies among the four drugs may adopt different definition. Therefore, the ambiguity creates challenge in comparing four drugs-studies and drawing above definitive conclusions.

As the COVID-19 pandemic winds down, most of the global population has already been infected at least once, and people in many countries have been infected multiple times. For individuals already experiencing long COVID, reinfection seems to exacerbate their existing symptoms. One survey showed that 80% of long COVID patients reported that reinfection exacerbated some of their symptoms (Willyard, 2023). The increase in the death risk of patients with long COVID may lead to a further decline in life expectancy, which may wipe out decades of progress (Willyard, 2023).

Although it was recently reported that the symptoms of long COVID would recover to some extent after 2 years, reducing the incidence of PASC had become the focus and hotspot of clinical research (Mateu et al., 2023; Ledford, 2024; Phetsouphanh et al., 2024). Early intervention with small-molecule antiviral drugs, such as Metformin, Ensitrelvir, Molnupiravir, and Nirmatrelvir, can effectively prevent the occurrence of long COVID, reduce the incidence of long COVID, and thus hinder the secondary damage of SARS-CoV-2 to the human body.
